# Supplementary material for: Prevalence of Steinert’s Myotonic Dystrophy and Utilization of Healthcare Services: A Population-Based Cross-Sectional Study
Source: Healthcare (Basel). 2024 Apr 16;12(8):838. doi: 10.3390/healthcare12080838 (PMC11050373; doi:10.3390/healthcare12080838)
Supplement: Supplementary file 1 [file healthcare-12-00838-s001.zip › healthcare-2907249-supplementary.pdf]

Supplementary Material

† Collaborators/Membership of the DM1-CM Working Group

Name		email	Affiliation
**First**	**Last**		
Alfredo	Rosado Bartolomé	alfredorosado76@gmail.com	Comité Científico Orphanet-España
Carmen	Prior de Castro	carmen.prior@salud.madrid.org	Department of Genetics, Hospital Universitario La Paz, Madrid, Spain
Gerardo	Gutiérrez Gutiérrez	gerardo.gutierrez@salud.madrid.org	Department of Neurology, Hospital Universitario Infanta Sofía, Madrid, Spain
Maria José	Trujillo Tiebas	MJTrujillo@fjd.es	Department of Genetics, Fundación Jiménez Díaz University Hospital (IIS-FJD, UAM), Madrid, Spain
